# Parenteral Nutrition Additive Shortages: The Short-Term, Long-Term and Potential Epigenetic Implications in Premature and Hospitalized Infants

**DOI:** 10.3390/nu4121977

**Published:** 2012-12-07

**Authors:** Corrine Hanson, Melissa Thoene, Julie Wagner, Dean Collier, Kassandra Lecci, Ann Anderson-Berry

**Affiliations:** 1 School of Allied Health Professionals, University of Nebraska Medical Center, Omaha, NE 984045, USA; 2 Pharmacy and Nutrition Care Services, Nebraska Medical Center, Omaha, NE 984045, USA; E-Mails: mthoene@nebraskamed.com (M.T.); klecci@nebraskamed.com (K.L.); 3 Alegent Health Bergan Mercy Medical Center, 7500 Mercy Road, Omaha, NE 68124, USA; E-Mail: Julie.wagner@alegent.org; 4 College of Pharmacy, University of Nebraska Medical Center, Omaha, NE 986045, USA; E-Mail: dcollier@unmc.edu; 5 College of Pediatrics, University of Nebraska Medical Center, Omaha, NE 981205, USA; E-Mail: alanders@unmc.edu

**Keywords:** TPN, premature infant, nutrient deficiencies, neonate

## Abstract

Nutrition support practitioners are currently dealing with shortages of parenteral nutrition micronutrients, including multivitamins (MVI), selenium and zinc. A recent survey from the American Society of Enteral and Parenteral Nutrition (ASPEN) indicates that this shortage is having a profound effect on clinical practice. A majority of respondents reported taking some aggressive measures to ration existing supplies. Most premature infants and many infants with congenital anomalies are dependent on parenteral nutrition for the first weeks of life to meet nutritional needs. Because of fragile health and poor reserves, they are uniquely susceptible to this problem. It should be understood that shortages and rationing have been associated with adverse outcomes, such as lactic acidosis and Wernicke encephalopathy from thiamine deficiency or pulmonary and skeletal development concerns related to inadequate stores of Vitamin A and D. In this review, we will discuss the current parenteral shortages and the possible impact on a population of very low birth weight infants. This review will also present a case study of a neonate who was impacted by these current shortages.

## 1. Introduction

Product shortages in the healthcare system can have a significant impact on the care provided to patients. In the case of premature or ill infants, high nutrient needs and a lack of nutrient stores make the appropriate administration of nutrition essential. At birth, the clamping of the umbilical cord immediately disrupts the delivery of nutrients from the placenta, which places these infants into a situation that makes adequate provision of nutrients extremely important and challenging. Though the preferred method of feeding, enteral feedings are often delayed in the premature infant due to the fragility and medical complications associated with prematurity. Management of co-morbid conditions, such as patent ductus arteriosis, chronic lung disease and feeding intolerance frequently involve restriction of fluids and delays in enteral feeding advancement. As a result, these cases usually remain dependent on parenteral nutrition (PN) for the first few weeks of life; however, those with complications, such as necrotizing enterocolitis, may require sole PN for much longer time periods. In the infant with congenital anomalies, such as gastroschisis, trachea-esophageal fistula or bowel disease, feedings may not be feasible for weeks or even months. There is growing evidence that inadequate nutrition in the first few weeks of life results in growth failure and poor long-term neurodevelopmental outcomes [1,2]. Due to concern for current shortages of parenteral nutrition (PN) micronutrient additives, the purpose of this paper is to review the potential effect of these shortages on premature and newborn infants and to present a case study of nutrient deficiency resulting from this shortage. 

## 2. Current Shortages and TPN Management Strategies

According the American Society for Enteral and Parenteral nutrition (ASPEN), all PN products, aside from dextrose and water, have been in short supply at some point since the spring of 2010 (see Table 1) [3]. At the time of this writing, a number of PN products continue to be unavailable or in short supply. The current status of these and other drug products can be accessed at the Food and Drug Administration web site [4].

**Table 1:** Parenteral Shortages 2010–2012.

Amino Acids	Ascorbic Acid	Calcium Chloride
Calcium Gluconate	Chromium	Copper
Cyanocobalamin	Fat Emulsions	l-cysteine
Magnesium Sulfate	Multivitamins (MVI)	Phytonadione (vitamin K)
Potassium Acetate	Potassium Phosphate	Selenium
Sodium Acetate	Sodium phosphate	Trace Elements
Vitamin A	Zinc	

ASPEN has provided timely and comprehensive advice to clinicians for dealing with PN product shortages. Recommendations for conserving products during shortages of electrolytes, vitamins and minerals are included in Table 2.

**Table 2:** ASPEN Recommendations for Conservation of PN Products.

Consider oral or enteral administration Prioritize patients, saving supplies for those most vulnerable patients Eliminate adding injectable electrolytes/minerals to enteral nutrition products Minimize the use of additives to daily maintenance IV fluids Reevaluate replacement algorithms or treatment protocols Carefully evaluate alternative supplies of individual and multiple electrolyte products that are available, including standardized, commercially available PN products

In a recent survey conducted by ASPEN, 25% of respondents stated their hospitals are in short supply of pediatric multi-dose products, and 28% indicated they are in short supply of single-dose products, such as selenium. When asked what actions were being employed in response to these shortages, 70% reported giving multivitamins (MVI) only three times per week, 28% were giving half the recommended dose daily, 27% were giving no MVI and 9% were giving adult MVI to pediatric patients [3]. 

Product shortages are not new and have historically been documented to cause morbidity and mortality. The omission of vitamins from PN has been associated with death as far back as 1989, when three people at a large university medical center died from refractory lactic acidosis caused by receiving thiamine-deficient PN [5]. In 1997, three additional patients receiving thiamine-free TPN due to a national shortage developed lactic acidosis, and later, in 1998, a case report of Wernicke encephalopathy and beriberi due to a multivitamin shortage was published [6]. Due to the historical documentation of such adverse outcomes in adults, we sought documentation of the potentially adverse effects in high-risk, premature infants. 

## 3. Nutritional Needs of Preterm Infants and Potential Effect of Shortages of PN Components

*Proteins and Amino Acids*: Per kilogram, premature infants have among the highest protein needs of any population during the lifecycle. Adequate protein provision of up to 4–4.5 g/kg/day is required for very low birthweight infants to match intrauterine accretion rates and to promote appropriate growth [7,8]. Adequate growth during Neonatal Intensive Care Unit (NICU) hospitalization is essential to reduce the risk for negative outcomes associated with growth restriction, including chronic lung disease, cerebral palsy, neurological impairment and rehospitalization after discharge [3,9]. The early administration of parenteral protein helps to offset the nutritional deficits that begin with preterm birth and continue throughout the infant’s hospitalization [10]. Early administration of PN that meets protein goals has been associated with improvements in both short-term [11] and long-term growth [1]. In contrast, infants not receiving early administration of amino acids have been found to have suboptimal head growth at 18 months of age [1]. Restrictions in head circumference growth are especially concerning, given the association between poor head growth and decreased neurodevelopmental outcomes [12]. Likewise, goal provisions of both protein and energy during the first week of life may positively influence 18-month developmental outcomes [13]. Furthermore, shortages of individual amino acids can have a profound effect on premature infant outcomes. For example, supplementation of neonatal PN with cysteine has been associated with improved nitrogen balance, which may support appropriate growth for both weight and head circumference [14,15]. The addition of cysteine may also help to lower the pH of the PN mixture. This allows increased amounts of calcium and phosphorus to be provided with a decreased risk for mineral precipitation, further promoting adequate bone mineralization and growth [16]. Consequently, shortages of amino acid formulations impacting early and adequate provision of protein in this population can have significant long-term ramifications.

*Lipids*: Fat provision is essential for life, but most specifically for premature infants to promote normal growth and development. Fat is the most calorically-dense nutrient and aids in the development of cells, the eye and the brain. Without lipid provision to PN-dependent infants, essential fatty acid deficiency (EFAD) develops quickly as there are reports as early as the second day of life [17]. EFAD may result in failure to thrive, dermatitis, thrombocytopenia, susceptibility to infection and poor would healing [17]. Fat-free PN as the primary source of nutrition may further result in suboptimal calorie provision and associated growth restriction. Increased total caloric intake in the first week of life has been associated with improved Bailey MDI scores in a preterm infant population [13].

*Minerals*: In addition to macronutrient shortages, micronutrient shortages have the potential to significantly impact the health of infants. One PN additive that is commonly used among the premature infant population is calcium gluconate. Although higher in aluminum content than other available PN calcium salts, calcium gluconate is often the additive of choice due to its favorable solubility curves, allowing for higher calcium and phosphorus administration. Like protein, calcium and phosphorus needs in premature infants remain high. Approximately 80% of total body calcium, phosphorus and magnesium at birth are accrued within the last trimester of pregnancy. Consequently, infants born prematurely have inadequately mineralized bones and require higher weight-based provisions of calcium and phosphorus compared to infants born at term [8]. Adequate magnesium provision is also necessary to promote bone growth, as hypomagnesaemia may hinder calcium absorption. In addition, many common medications used for NICU infants can decrease calcium and magnesium absorption, including diuretics, steroids and anti-convulsants [8,18]. It has been well-documented that premature infants receiving inadequate amounts of calcium and phosphorus from PN have higher incidences of osteopenia and elevated levels of serum alkaline phosphatase, which may indicate poor bone mineralization and rapid bone turnover [19]. Clinical manifestations of poorly mineralized bone will vary depending on the degree of bone mineralization. Bone fractures may develop in these infants as a result and may negatively affect the stability of the chest wall, which can lead to atelectasis or an increased risk of chronic lung disease [14]. 

*Trace Elements*: Only two trace elements are recommended on the initial day of PN; zinc and selenium. Other trace elements are not thought to be needed until after two weeks of age [14]. Zinc is a cofactor in more than 300 metabolic processes and supports protein and nucleic acid synthesis, immune function, gene expression and growth. Two-thirds of the total body zinc is transferred from mother to fetus during the last trimester of pregnancy and serum levels of zinc decline progressively in infants on zinc-free PN [20]. As a result, ill neonates are at a high risk of developing zinc deficiency if adequate supplementation cannot be provided. Additionally, most commercially-available trace element formulations contain insufficient zinc to meet the needs of the preterm infant. Therefore, supplemental zinc must be added to PN, creating an additional mechanism for shortages. 

Selenium is a component of glutathione peroxidase, a compound which protects against free radical injury. Premature infants are often exposed to high levels of oxygen and resulting oxidative stress. It has been speculated, though not proven, that selenium could be an important contributing factor in the pathogenesis of disorders, such as bronchopulmonary dysplasia [21]. Most of the placental transfer of selenium occurs after the 36th week of gestation [20]; hence, most premature infants will be born selenium-depleted. Serum selenium concentrations have been shown to decrease quickly in infants treated for respiratory distress syndrome who receive selenium-free or selenium-deficient PN [22]. A 2009 Cochrane review of selenium supplementation suggested that the currently recommended dose of selenium (2 µg/kg/day) for preterm infants receiving parenteral nutrition is inadequate to maintain selenium concentrations, so a dose of 3 µg/kg/day may be more appropriate to achieve concentrations found in healthy breast-fed infants [23]. As current shortages make it difficult to provide even the previously recommended amounts, implementing new evidence-based recommendations for increased nutrient dosing becomes nearly impossible.

Inadequate provision of many other trace elements also poses nutritional risks for premature infants. For example, both rapid growth and limited stores make premature infants at risk for copper deficiency, which can result in osteoporosis, neutropenia or hypochromatic anemia [17]. Like zinc and selenium, accretion of these nutrients occurs primarily during the last trimester of pregnancy, so adequate provision is essential.

*Vitamins*: Premature infants require higher amounts of most vitamins when compared to term infants, due to increased nutrient losses or increased requirements for growth. Recent studies have documented nutrient deficiencies, specifically vitamin D deficiency, are highly prevalent in preterm births [24,25,26,27]. Vitamin D supplementation during infancy is associated with increased bone mass in childhood [28]. Consequently, deficiency and inadequate supplementation may result in rickets or craniotabes [29]. Recent research has also expanded our understanding of vitamin D status during infancy to include health benefits beyond bone status. Poor vitamin D status during early infancy is now thought to be associated with other negative health outcomes, such as type 1 diabetes [30,31], and pulmonary problems, such as respiratory tract infections or wheezing later in childhood [32,33,34]. The current recommendation for parenteral vitamin D intake is 400 IU/day [8] which is the amount included in 5 mL in most pediatric PN MVI formulations. Concerning, however, is that PN pediatric MVI dosing is weight-based according to the manufacturer instructions; so many low birth weight infants receive less than 5 mL (400 IU Vitamin D) daily. These dosing practices, combined with product shortages, may make adequate administration of vitamin D challenging in infants dependent on PN. Monitoring the vitamin D status of newborns is additionally challenging, as 25(OH)D levels are not an accurate marker of vitamin D status in the newborn population. In 2006, Singh *et al*. showed that a biologically inactive C-3 epimer of vitamin D is present in high levels in the bloodstream of infants up to one year of age and may lead to an overestimation of vitamin D status in infants and concerns regarding vitamin D overdosing in others [35]. 

Administration of vitamin E is recommended immediately after birth in premature infants, as some older studies suggest a reduction in incidence and severity of intraventricular hemorrhage. A plasma target of >1 mg/dL by 24 h of life, and 2 mg/dL by three days of life has been recommended to reduce IVH [14,36,37]. These levels should be attainable with the administration of current PN MVI formulations, which would provide 2.8 mg/kg/day of vitamin E if given daily. However, limiting the amount of MVI provided to premature infants, as many institutions are now being forced to do, could negatively impact serum levels. 

Vitamin A is necessary in premature infants for normal lung growth and for maintaining the integrity of the respiratory epithelial track. Preterm infants are born with essentially no hepatic reserves of Vitamin A, so levels of vitamin A at birth are reflectively low [38]. Therefore, Vitamin A needs to be provided consistently from PN to promote adequate intake. However, a 2011 study demonstrated that despite a policy to introduce intravenous-lipid supplemented PN by day of life two, only 11% of infants consistently met the recommended daily intake of vitamin A during the first two weeks of life [39]. Considering inadequate provision despite the best efforts, it remains concerning that many PN-dependent infants are receiving reduced MVI dosing due to current shortages. The role of vitamin A in preventing neonatal chronic lung disease has consistently been documented, including evidence that intramuscular supplementation of vitamin A in extremely low birthweight infants improves respiratory outcomes [38,40]. A meta-analysis of low birthweight infants demonstrated that either enteral or parenteral supplementation with vitamin A reduced the risk of both death and oxygen requirement at one month of age, and the risk of oxygen requirement at 36 weeks corrected gestational age [38]. This practice may be limited, since intramuscular injections are considered painful; however, current product shortages of the vitamin injections would inhibit this practice from being initiated in NICUs targeting bronchopulmonary dysplasia (BPD). In addition to its role in lung integrity, vitamin A appears critical in the development of ocular photoreceptors. During the third trimester of pregnancy, the ocular content of rhodopsin (of which vitamin A is an essential component) increases 10-fold [39]. For the NICU hospitalized infant, an adequate substrate in the form of vitamin A needs to be available to support this process in the extrauterine environment. Recommended PN administration of vitamins and minerals discussed above are given in Table 3. Vitamin levels provided by current MVI dosing guidelines are provided in Table 4. 

**Table 3:** Recommended PN Administration of Select Nutrients *.

	Birth weight <1.5 kg	Term Infants
Calcium	3 mEq/kg/day	2 mEq/kg/day
Zinc	1000–3000 μg/kg/day	250 μg/day <3 months
		100 µg/day >3 months
Selenium	1.3–4.5 μg/kg/day	2.0 μg/day
Vitamin A	700–1500 IU/day	2300 IU
Vitamin D	400 IU/day	400 IU/day
Vitamin E	6–12 IU/kg/day	7 IU/day

* Adapted from [16].

**Table 4:** Vitamin Levels Provided by Current Dosing of Pediatric MVI.

	Per 1.5 mL MVI (<1000 g)	Per 3.25 mL MVI (1001–2500 g)	Per 5 mL MVI (>2500 g)
Vitamin C (mg)	24	5.2	80
Vitamin A (IU)	690	1495	2300
Vitamin D (IU)	120	260	400
Thiamine (B1) (mg)	0.36	0.78	1.2
Riboflavin (B2) (mg)	0.42	0.91	1.4
Pyridoxine (B6) (mg)	0.3	0.65	1
Niacin (mg)	5.1	11.1	17
Dexpanthenol (mg)	1.5	3.3	5
VitaminE (IU)	2.1	4.6	7
Vitamin K (mg)	0.06	0.13	0.2
Folic Acid (μg)	42	91	140
Biotin (μg)	6	13	20
B12 (μg)	0.3	0.65	1

*Epigenetic Ramifications of Early Neonatal Nutrition*: In addition to the acute problems from inadequate nutrient provision during the NICU stay, accumulating evidence implies that the early prenatal period is critical for influencing disease risk for a range of disorders that may develop later in life. There is considerable evidence suggesting that the risk of developing non-communicable diseases can be altered by modifying influences of early-life environment, which includes nutritional factors. This programming appears to be largely independent of genomic sequences and is likely to be mediated by epigenetic mechanisms. Epigenetic programming during development is widely believed to have a long-lasting effect on the health of a newborn [41]. Epigenetic changes are modification to the DNA, which can be caused by various factors, including the environment, which do not change the underlying sequence. 

Studies have demonstrated that infants born prematurely have decreased insulin sensitivity when compared with those born at term [42,43]. In one study of preterm *vs.* term infants and insulin sensitivity, all of the infants born prematurely had inadequate protein intake in the first month of life, which was proposed as a trigger to an epigenetic modification of genes involved in glucose regulation [42]. Supplementation with vitamin D during infancy has been reported to confer protection against beta-cell regulated autoimmune disorders [44,45]. A potentially very important activity of vitamin D receptor agonists is their capacity to induce *in vitro* and *in vivo* tolerogenic dendrite cells, which are able to enhance suppressor T-cells that, in turn, inhibit T-cell responses. Failure of tolerance mechanisms may lead to auto-reactive T-cell activation and to induction of autoimmune diseases [46]. In a birth-cohort study published in 2001, Hypponen *et al*. found that vitamin D supplementation during the first year of life is associated with a lower risk of type-1 diabetes [47]. This prospective study followed 12,055 infants born in Finland and collected data in the first year of life regarding vitamin D supplementation practices; later in life, data was collected on the diagnosis of type I diabetes. At this time in Finland, the recommended level of Vitamin D supplementation for infants was 2000 IU daily, and infants in the study were classified as follows: receiving daily supplementation of less than 2000 IU, within 2000 IU or over 2000 IU. Higher vitamin D supplementation in infancy was associated with a decreased frequency of Type 1 diabetes later in life when adjusted for neonatal, anthropometric and social characteristics. Infants who regularly took the recommended dose of 2000 IU vitamin D per day had a relative risk (RR) of 0.22 (95% CI: 0.05–0.89) compared to those who regularly received less than the recommended amount. Children suspected of having rickets during the first year of life had a RR of 3.0 (95% CI: 1.0–9.0) compared with those without such a suspicion [31]. These results are consistent with the findings of the EURODIAB study in 1999, which was a large multicenter and population-based case-control study on early risk factors of childhood onset Type I diabetes. Vitamin D supplementation in early infancy to prevent rickets in seven different European regions was also associated with a decreased risk for childhood-onset, insulin-dependent diabetes. The favorable association with vitamin D supplementation persisted after adjustment for birth weight, duration of breast feeding, maternal age and study center [30]. Additionally, vitamin D supplementation in infancy has also been associated with a decreased risk of pre-eclampsia later in life [47]. Consideration of the potential for early nutrition interventions to have long-term health outcome implications is crucial when designing nutrition support regimens for the premature infant population.

*Potential Cost of Shortages*: Poor outcomes attributable to PN shortages can quickly become costly to both an infant’s health and associated healthcare costs. In 2007, the Journal of Pediatrics compiled data regarding health care costs associated with initial hospitalization for premature infants. The data clearly showed a monetary cost increase for all premature births compared to term births, but this cost was four-to-seven-times higher for low birthweight infants with complications like chronic lung disease or intraventricular hemorrhage. Additionally, 15% of all premature or low birthweight infants were given a diagnosis of slow growth, malnutrition or small for gestational age. The cost for these categorized infants was nearly $450 million [48]. Despite high health care costs, complications associated with inadequate nutrition from PN shortages can have a much more devastating toll on infant health. Complications, such as intraventricular hemorrhages or developmental delay, may impact life-long ability and care requirements. Similarly, chronic diseases pose increased risks for infections and infant mortality. Adequate provision of nutrients from PN is essential to help preserve the health integrity of these infants, but unfortunately, PN shortages have the potential to do the opposite.

*Case Study*: At our care facility, one infant on prolonged PN-developed selenium deficiency with associated skin symptoms. This infant was born at an estimated gestational age of approximately 26 weeks with a birth weight of 790 g. The infant developed a bowel perforation within the first few weeks of life and required surgery, with a resulting jejunostomy placement. The infant required both sole and partial PN for most of his extensive hospital stay, and was approximately four months of age when able to take full enteral feedings. Due to current shortages, no selenium was available to add to the PN formulation. The infant developed dry, scaly skin and a selenium level was checked around three months of life secondary to skin symptoms and recent PN additive shortages. The selenium level was reported from the laboratory as 11 μg/L, which is well below the normal range for serum selenium of 23–190 μg/L. Conditions, such as macrocytosis and fatal cardiomyopathy, have been reported when selenium levels fall below 16 μg/L [23]. Once enteral feedings were established, 25 µg of oral selenium was supplemented every other day, because no parenteral selenium was available. By discharge around five months of life, the infant was on calorically-dense full enteral feedings and his skin symptoms had resolved, so at this point selenium supplementation was discontinued. 

*Potential Solutions*: As is evident from Table 2, whenever possible during a shortage of PN products, prioritization of resources is crucial in ensuring that the most vulnerable populations receive critical products. Additional strategies to potentially maximize the nutrition status of premature infants during PN product shortages should be explored. For instance, our care facility was forced to reduce pediatric PN MVI to tri-weekly provision to conserve supplies. With the concern for inadequate nutrient provision to our extremely and very low birthweight infants, we increased the weight-based MVI dosing. For example, infants receiving 1.5 mL of PN MVI would receive 3.25 mL tri-weekly. Though this is not a documented strategy, it does allow for a higher administration of nutrients while conserving available local resources.

## 4. Conclusion

This review demonstrates the potential short-term and long term epigenetic impacts that current PN shortages can have on premature and ill infants. Clinicians must, therefore, make decisions using current evidence and clinical judgment to ensure avoidance of poor outcomes in situations where adequate administration of all essential nutrients is not possible. 
